# Integrating nutrition and obesity prevention considerations into institutional investment decisions regarding food companies: Australian investment sector perspectives

**DOI:** 10.1186/s12992-022-00885-7

**Published:** 2022-11-08

**Authors:** Ella Robinson, Christine Parker, Rachel Carey, Anita Foerster, Miranda R Blake, Gary Sacks

**Affiliations:** 1 Global Centre for Preventive Health and Nutrition (GLOBE), Institute for Health Transformation, Deakin University, Geelong, VIC Australia; 2 Melbourne Law School, The University of Melbourne, Melbourne, VIC Australia; 3 School of Agriculture and Food, The University of Melbourne, Melbourne, VIC Australia; 4 Monash Business School, Monash University, Melbourne, VIC Australia

**Keywords:** Responsible investment, Environmental Social Governance, Obesity, Food industry, Accountability

## Abstract

**Background:**

There is growing recognition that current food systems are both unhealthy and unsustainable, and are increasingly shifting toward the supply and marketing of unhealthy, ultra-processed foods and beverages. Large food companies hold substantial power within food systems and present a significant barrier to progress on addressing issues related to nutrition and obesity prevention. Institutional investors (such as pension funds) play a key role in influencing corporate governance and practices, and are increasingly incorporating environmental, social and governance (ESG) considerations within investment decisions. By considering nutrition and obesity prevention, institutional investors present a potential avenue for driving increased food industry accountability for their population health impact. This study investigated views of stakeholders in the Australian investment sector on the incorporation of nutrition and obesity prevention considerations within institutional investment decision-making regarding food companies.

**Methods:**

Fifteen in-depth, semi-structured interviews were conducted in 2020-21. Participants were predominantly Australian-based, and included representatives from asset management companies, superannuation funds, ESG advisory/consultancy firms, ESG research providers, and relevant advocacy groups. Interviews examined challenges and opportunities to the integration of nutrition and obesity prevention considerations within institutional investment decision-making. Interviews were analysed using deductive thematic analysis, informed by a theoretical change model.

**Results:**

Several participants reported that their institution factored nutrition and obesity prevention considerations into their investment decisions; however, attention to nutrition-related issues was limited, generally perceived as ‘niche’, and not yet institutionalised. Key challenges and opportunities were identified at the employee, investment organisation, investment sector, government and non-government levels. These challenges and opportunities centred around experience and knowledge, quality and availability of ESG data and benchmarks, importance of investor coalitions, and demonstration of financial risks related to nutrition and obesity.

**Conclusion:**

There are a range of steps that could be taken to help ensure more systematic and effective consideration of issues related to nutrition and obesity prevention within institutional investment decision-making in Australia, including: (1) improved nutrition-related reporting metrics and benchmarking criteria for food companies; (2) better articulation of the financial risks that unhealthy diets and obesity pose to investors; (3) enhanced investor advocacy on unhealthy diets and obesity through investor coalitions and; (4) detailed guidance for investors on how to address unhealthy diets and obesity. Better engagement between the Australian public health community, institutional investors and government regulators is critical to drive changed investor practice in this area.

**Supplementary Information:**

The online version contains supplementary material available at 10.1186/s12992-022-00885-7.

## Background

There is growing recognition that current food systems are both unhealthy and unsustainable and are increasingly shifting towards the supply and marketing of unhealthy, ultra-processed foods and beverages [1–3]. These changes to the food supply, coupled with low levels of physical activity, have been identified as the primary driver of the high rates of obesity globally [4, 5]. Unhealthy diets and obesity are now among the leading risk factors for death and disability worldwide [6]. The economic and societal costs of obesity are wide reaching, with approximately 13% of all global healthcare expenditure attributed to obesity [7]. As evidenced through the COVID-19 pandemic, human health and wellbeing is central to a healthy and sustainable society and economy [8]. In order to achieve societal and economic prosperity, improvements to the healthiness of food systems are urgently needed.

In an effort to encourage increased efforts to address unhealthy diets and obesity, the World Health Organization has set a range of global nutrition and obesity prevention targets [7, 9]. The United Nations Sustainable Development Goals (SDGs) also include several nutrition-related objectives, such as SDG 3 (good health and well-being) and SDG 2 (Zero Hunger), that call for reduced non-communicable disease mortality and an end to malnutrition in all its forms [10]. However, actions to address the healthiness of food systems have been stalled by a number of factors, including a lack of political commitment to obesity prevention, challenges in implementing policies that impact multiple sectors, and limited co-ordination from civil society [11, 12]. Food industry opposition has also been identified as presenting substantial obstacles to policy and regulatory change for improving population diets and addressing obesity [11, 13]. Due to globalisation and the increasing market concentration of some sectors of the food industry in many countries and regions, a small number of large corporations now hold substantial power and influence within food systems [14, 15]. Several authors have identified the negative implications for public health that have resulted from such power and influence [16–19].

The food industry in Australia, like many other high- and middle-income countries, is highly concentrated [20]. In particular, the Australian food retail market is one of the most concentrated in the world [16, 20], with two retailers (Coles and Woolworths) controlling more than 65% of the grocery market share [21]. Moreover, the food and beverage manufacturing sector is largely dominated by a small number of multi-national companies who control the food supply and hold substantial levels of market power [22]. In this context, increasing food company accountability for their role in supplying and marketing unhealthy products, as well as driving improvements in nutrition-related policies and practices, forms an important component of efforts to improve population diets in Australia and globally. There are a number of areas where food companies can improve their policies and practices to facilitate healthier population diets. These include setting targets to increase the proportion of sales from healthier products, improving the healthiness of food products, restricting exposure of children to unhealthy food marketing, and improving nutrition labelling [15, 23]. Alongside this, there is an increasing push for greater transparency from food companies on their nutrition-related policies and practices, with several recent initiatives focused on monitoring and evaluating progress over time [14, 24].

Institutional investors (e.g., superannuation/pension funds, mutual funds, insurance companies, asset management companies) can play a key role in influencing corporate governance and practices [25–27], through decisions to invest in or divest from stocks and by exercising their ownership rights to influence investee companies. This includes voting for and against company decisions (e.g., executive remuneration, election of company directors) and engaging with companies on various issues [27, 28]. As part of their investment decision-making, institutional investors are increasingly factoring in environmental, social and governance (ESG) considerations (responsible investment) [29, 30]. Where Corporate Social Responsibility (CSR) represents a business’ voluntary actions to positively impact society [31], ESG is coming to the fore as a way of measuring and quantifying business performance through criteria-led metrics [32]. Responsible investment activities often reflect financial goals, such as mitigating financial risks associated with ESG issues and supporting sustainable profit growth [33]. Financial regulators and finance-sector industry associations increasingly recognise ESG considerations as financially material and, accordingly, that ESG considerations should be integrated within investment decisions in line with fiduciary duties owed to investment beneficiaries [34]. Furthermore, international evidence suggests that institutional investors can influence corporate practices related to ESG concerns [35, 36] and improve the ‘social’ performance of companies [25].

In 2021, the Responsible Investment Association of Australasia (RIAA) reported that responsible investment assets represent more than 40% of total assets under professional management in Australia (over AUD$1,281 billion) [37]. The value of responsible investments is growing rapidly, and Australia’s superannuation (pension) market (responsible or otherwise) has one of the highest growth rates in the world [34]. This means there is substantial opportunity for Australian investors, particularly superannuation funds, to use their influence to improve the practices and performance of food companies.

For institutional investors, integrating considerations related to nutrition and obesity prevention as part of ESG practices can mitigate risks and harness benefits associated with healthier diets [38, 39]. Regulatory risks are increasing for the food industry, with over 40 countries globally implementing some type of tax on sugary drinks [19] and an increasing number of governments introducing regulations on marketing to children, labelling, and promotion/placement of unhealthy products [40, 41]. Investor advocacy groups are increasingly demanding accountability from food companies for their nutrition-related actions [42–44], and investors and companies are responding to trends and growth opportunities related to consumer preferences for healthier and more sustainable products [45, 46]. Food companies that are ill-equipped or unwilling to improve their nutrition-related policies and practices are therefore exposed to substantial earnings risks. Furthermore, institutional investors with longer-term investment horizons and diversified portfolios may stand to benefit from the positive societal and economic impacts associated with a healthier society [38].

Previous research suggests that investors in Australia are not considering nutrition or obesity in comprehensive or systematic ways [47, 48]. For example, a 2020 study examining publicly available information from 35 Australian asset managers and superannuation funds engaged in responsible investment, found that whilst 18 out of the 35 investors reported using responsible investment strategies that incorporated nutrition- and obesity-related considerations, comprehensive action was lacking [48]. When investors did address nutrition and obesity, they rarely reported engaging with food companies around improvements to nutrition-related policies and practices (marketing to children, reformulation, lobbying), or including or excluding food companies in investment portfolios based on the healthiness of their products [48]. This previous study identified the need for further research that engages directly with the investment sector to understand how and why issues related to nutrition and obesity prevention are considered within investment decision making in Australia. This study aimed to explore challenges and opportunities related to the consideration of nutrition and obesity prevention within institutional investment decision-making regarding food companies, from an Australian perspective.

## Methods

### Study design

This qualitative study used deductive thematic analysis of in-depth semi-structured interviews with stakeholders working within the institutional investment sector. Stakeholders were predominantly based in Australia.

### Sample

The initial participant sample was a purposively recruited sample of participants able to provide in-depth insight into the study aims due to their experience working within or with the institutional investment sector. The sample of organisations selected for inclusion in this study (e.g., institutional investment organisations, ESG research providers, NGO and advocacy organisations) was informed through previous studies [48, 49]. Contact details were identified using publicly available information (e.g., through members of the Responsible Investment Association of Australasia).

### Data collection

In-depth semi-structured interviews were conducted with fifteen participants by video conference (using Zoom) between May 2020 and February 2021. The interview schedule (Additional file 1) was developed based on the findings and recommendations from previous desk-based reviews conducted as part of a broader research program investigating investment in healthy and environmentally sustainable food systems [48, 49]. The interview schedule included the following topics: the extent to which healthy and environmentally sustainable food systems are considered and prioritised; drivers of considerations related to healthy and environmentally sustainable food systems; data, metrics and reporting on healthy and environmentally sustainable food systems; challenges and opportunities of responsible investment that incorporates healthy and environmentally sustainable food systems; and ways that researchers could support action in this area. The focus of this paper was on concerns related to unhealthy diets and obesity prevention in Australia, particularly as they relate to investment in food companies. Analysis of a broader range of environmental sustainability considerations will be reported in a separate publication.

Written consent was obtained from each participant (ethical approval for this project was granted by the Deakin University Human Ethics Advisory Group - Health, approval number HEAG-H 137_2019). Interviews were audio recorded, with the participant’s permission. Interview transcripts were transcribed verbatim and stored in de-identified format, with participant identification codes stored separately. Data collection was iterative, and ‘saturation’ - the point at which no new themes emerged from the interview data [50] – was estimated to have been reached between interviews 12 to 15.

### Data analysis

Data were analysed using deductive thematic analysis. The initial coding framework was guided by a social change theoretical model [31] as well as the authors’ existing knowledge of this topic [51], with openness to emerging codes. Aguilera et al.’s 2007 [31] social change theoretical model incorporates theories of organisational change, corporate governance and capitalism to understand what catalyses business organisations to engage in Corporate Social Responsibility (CSR) initiatives that impact ‘social change’ [31]. The model contends that organisations are pressured to engage in CSR by different actors who use various mechanisms to encourage or discourage CSR, and that it is helpful to investigate the roles of these actors operating at different levels, such as internally within an organisation, across the broader sector, and at the regulatory level. This model was chosen because of its specific focus on social change as the outcome of CSR initiatives, which aligns to framing responsible investment as a potentially important contributor to improved population diets. In this context, CSR is undertaken by institutional investors to influence investee companies, whereby improvements to corporate practices and performance may impact population nutrition outcomes. The model was adapted for use in this study to focus on what challenges and opportunities exist to drive and support the consideration of issues related to nutrition and obesity prevention within this process. The relevant actors considered as part of the analysis were grouped as follows: employees within institutional investment organisations, institutional investment organisations, investment sector (including ESG research providers, international finance organisations, peak industry bodies, market infrastructure and facilitation), governments, and non-government organisations. Refer to Additional file 2 for the coding framework.

All data was analysed in NVivo 12. One researcher (ER) analysed all transcripts, and another researcher independently analysed a sample of initial interviews to check for consistency in codes. Consistency between codes was cross-examined and discrepancies were resolved between coders. Codes were grouped according to the dominant themes.

The results section firstly describes the characteristics of participants included in this study, then outlines challenges and opportunities for increased consideration of issues related to nutrition and obesity prevention within institutional investment decision-making in Australia. In line with our theoretical model, challenges and opportunities were organised according to the actor group to which they relate (i.e., employees within investment organisations, investment organisation, investment sector, government, non-governmental). We present challenges and opportunities together, under each actor group heading, as these were often interrelated. The discussion section synthesises these findings to present key recommendations for action.

## Results

### Participant characteristics

Fifteen in-depth interviews were conducted. Participants included: institutional investors (asset management, superannuation) (n = 7, three of which had an ethical values focus); ESG advisory/consultancy firms (n = 2); representatives of investment industry groups (n = 2); ESG research providers (n = 2); and non-governmental organisations (NGOs) working with the investment sector with a focus on food systems and/or health related issues (n = 3). One organisation was counted as both an asset management and ESG advisory/consultancy firm. Participant roles were predominantly high-level management positions (such as Chief Investment Office or, Head of ESG/responsible investment) (n = 11), as well as project management and research analyst roles (n = 4).

### Key challenges and opportunities

Based on the interviews conducted, we identified a range of key challenges and opportunities for increased consideration of issues related to nutrition and obesity prevention within institutional investment decision-making in Australia (Table 1). The challenges and opportunities as they relate to each actor are discussed, in turn, below.

**Table 1:** Actor level challenges and associated opportunities to the consideration of issues related to nutrition and obesity as part of institutional investment decision-making

Actor	Theme	Challenges	Opportunities
**Employees within investment organisations**	Experience and training	• Level of experience and training regarding ESG investment	
	Internal pressure/buy in		• Employee interest/passion for addressing ESG issues, including nutrition- and obesity-related issues
**Investment organisations**	Competing duties and issues	• ESG ‘issue overload’ makes it difficult for food systems related issues (including obesity) to be high on the agenda • Perception that addressing ESG issues conflicts with fiduciary duty	
	Extent of topic-specific knowledge	• Limited understanding of issues related to nutrition, obesity prevention and food systems • Difficulties in defining what healthy food systems look like • Complexities around addressing nutrition and obesity as an ESG issue (not clear cut like other ESG issues, e.g., tobacco)	• Increase knowledge of what best practice looks like • Frame issues related to nutrition and obesity prevention using the SDGs • Harness opportunities to frame nutrition and obesity prevention as an ESG issue as responsible investment shifts from niche to mainstream
	Investment ethos and approach	• Many institutional investors think about ESG in terms of financial impact only (i.e., simplistic and superficial terms) • Philosophical view amongst investment sector that environmental issues are easier to address (and more important) than social issues • External management of funds (e.g., superannuation funds that outsource to asset managers) can narrow ESG focus	• A longer-term investment horizon can facilitate consideration of ESG issues (e.g., unhealthy diets and obesity) • Universal ownership (i.e., highly diversified portfolio) can increase attention to ESG issues • Active management style (versus passive management style) can facilitate ESG engagement and portfolio selection • Ethical values (e.g., exclusion of certain industries based on ethical views) can override financial imperative to support ESG goals
	Exposure to food companies	• Limited opportunities to invest in and engage with food and agricultural companies on the Australian Securities Exchange (ASX) • Perception that issues related to nutrition and obesity prevention are only relevant to multinational food companies • Australian investors have less power to exert ownership rights over international equities (e.g., food companies)	• Highlight investment opportunities in healthier food companies • Impact investing / venture capital can enable investment in positive food-related ventures
	Member/client demand	• Lack of demand for addressing ESG issues by superannuation fund members/clients • Lack of demand for responses to obesity-related ESG issues by superannuation fund members	• Client/member demand can increase investor attention to ESG issues (e.g., unhealthy diets and obesity)
	Brand reputation	• Narrow focus on ‘headline’ issues that capture public and government attention	• Investors may follow what others are doing to address ESG issues (herding behaviour) • Competitive advantage can be gained from demonstrating leadership on ESG themes
	Demonstration of financial risks	• Lack of clarity around the link between food systems related issues (including nutrition and obesity) and financial performance	• Need for further demonstration of financial risks (regulatory, legal, reputational) associated with ESG issues, including obesity • Potential for framing regulatory risk as ‘real’ rather than ‘hypothetical’
**Investment sector**	Quality and availability of ESG data	• Shortcomings (e.g., inconsistent and inadequate data) of currently available ESG data and ratings • Lack of quality benchmarks and data on food systems issues	• Increase uptake and use of appropriate food-related metrics by ESG research providers • Increase understanding of how to compare and measure food company ESG impact and exposure • Need for benchmarking and accountability initiatives that draw attention to food-related issues
	Focus on systemic issues/crises	• Lack of systemic thinking around food systems related issues and responsible investment • Perception that the links between healthy and sustainable food systems and climate change are not well established	• Systemic risks can spur action on ESG issues • Opportunities to highlight the systemic nature of obesity • COVID-19 is an entry point for discussion around health • Ability to demonstrate the links between climate change and food systems
	Exercising voice through collection action		• Investor involvement in political advocacy and public policy engagement can facilitate consideration of ESG issues • Co-ordinated engagement with other investors/ advocacy groups can enhance advocacy
**Governments**	Regulatory measures and legal constraints	• Perceptions about legal constraints to considering ESG in investment decision making	• Obesity-related regulatory measures in Australia and internationally can raise primacy of issue amongst investors • Regulatory changes and legislation to address ESG issues can spur investment action on particular issues
	Resourcing and leadership	• Lack of governmental leadership to address food systems issues in Australia • Few governmental resources dedicated to food systems issues in Australia • Australian government vocal against ESG-related engagements by investors	• Government inaction on ESG issues may prompt the investment sector to act • International government requirements and conventions (e.g., EU taxonomy) can strengthen action on ESG issues
**Non-government organisations**	Media attention and public opinion		• Level of controversy associated with particular ESG issues triggers action • Level of attention to ESG issues in the media can increase attention to ESG issues • Public opinion can facilitate the consideration of ESG issues by investors
	Advocacy	• Limited groundswell on food systems related issues in Australia • Difficult for small advocacy initiatives to demonstrate the cumulative risk of food systems related ESG issues (e.g., unhealthy diets and obesity)	• Advocacy groups can promote action on food systems issues • Emotive stories can help to convey the importance of addressing ESG issues to investors • Activities of watchdogs and advocacy groups can promote accountability

ESG = environmental, social, governance; EU = European Union; SDGs = Sustainable Development Goals

### Employees within investment organisations

Participants with broad insight into the investment industry (i.e., from investment industry groups, ESG research providers and ESG advisory/consultancy firms) identified that a key challenge faced across the responsible investment sector was the lack of experience in ESG amongst employees within investment organisations. These participants noted that responsible investment was, until recently, a fringe concept only applied by a small number of specialised investors. Due to the rapid expansion of responsible investment within, so called, mainstream finance, some participants pointed out that the training and capacity of those working in investment institutions, including those in dedicated ESG teams, was sometimes not up to the task:

*“The ESG world [has] grown very quickly and the skillset of the community is nowhere near up*.

*to the task…. The training is not there, we don’t have decades or generations of capacity building*.

*behind us.” – participant (ESG research provider)*.

In regard to consideration of issues related to nutrition and obesity prevention specifically, participants from a range of organisations indicated that there was a general lack of knowledge around health and nutrition issues related to food systems. However, several participants reported that an opportunity for increased action was an employee’s personal interest or passion for ESG issues, which through internal buy-in could help to increase attention on particular ESG areas within an investment organisation:

*“I didn’t want our standard fund to invest in [meat producer]. I hate factory farming and so I made it my mission to come up with a way of clearly demonstrating the link between factory farming and earnings risks.” – Participant (asset management company)*.

### Investment organisations

A number of organisational mechanisms were reported to hinder responsible investment by participants. The sheer amount of ESG issues competing for investor attention was seen as impeding the prioritisation of issues like obesity within investment decisions. Participants from a range of organisations talked about how literacy around how to define and address nutrition and obesity-related issues was lacking, and that there was a substantial level of complexity associated with incorporating nutrition and obesity-related issues to a portfolio when compared to other ESG issues like climate change.

*“It’s much more eclectic kind of reasons why animal welfare, or nutrition for example hits investors’ interest, and it’s relatively company specific. Climate change can apply to just about every company, in one way or another. Whereas nutrition is probably a little bit more difficult to do that. What does nutrition mean to a bank? … I think that’s part of the reason why it [nutrition] probably hasn’t had the same sort of profile [as a consideration for institutional investors].” – Participant (ESG advisory/consultancy firm)*.

A number of participants noted that the investment ethos and approach of the organisation could dictate the extent to which ESG issues were considered. Participants commented that institutional investors were generally thinking about ESG in terms of financial impact only, whereby companies that rate poorly on ESG performance but high on profit growth were still seen as attractive investment options, and ESG was not considered in a systemic way (e.g., only one of many considerations factored into investment analysis, use of high-level data on company ESG performance and focus on single companies). Participants explained that a longer-term investment horizon and highly diversified portfolio may facilitate consideration of obesity-related issues, because managing ESG risks and opportunities was perceived as promoting sustainable returns across a broad range of companies in the long run. Additionally, those investors with less diversified portfolios, but a more active investment approach (versus passive approaches that invest in the whole market) was seen as facilitating the consideration of obesity-related issues through in-depth analysis of a smaller portfolio of companies and a willingness to engage. A small number of participants from investment organisations with ethical values also reported that ESG goals could take precedence over wealth maximisation for their organisation.

Participants commonly reported that there were limited opportunities to invest in food companies on the Australian Securities Exchange (ASX). Some participants noted that there was a perception that obesity-related issues were more relevant to large, multinational food companies and that Australian investors had less power to exert ownership rights over internationally listed equities. Several participants commented that Australian investment organisations were therefore likely to focus their engagement efforts on locally listed companies, and the lack of ASX-listed food companies within their portfolios may have contributed to the limited focus on obesity-related issues to date.

*“I think why this issue probably hasn’t got the focus in Australia in particular is that a lot of the day-to-day work [for investors] is going and talking to companies we invest in. In the ASX [Australian Securities Exchange] there are probably not that many companies in this area [food companies]. It [nutrition] is not as readily accessible as other [ESG issues], just because the representation in the market, at least in the publicly listed market in Australia, isn’t as great.” – Participant (superannuation fund)*.

The majority of participants from investment organisations pointed out that a key facilitator of responsible investment generally, and consideration of issues related to nutrition and obesity prevention specifically, was client or superannuation fund member demand. Participants noted that many asset management company and superannuation funds were now under pressure to address ESG issues from their clients and members. Furthermore, participants from superannuation funds noted that they were likely to cater their consideration of ESG issues towards the needs of their particular member base (e.g., a particular industry or ethical view). A small number of participants reported that external pressure from the public could influence the extent to which investors focus on ESG issues, particularly where not addressing particular issues could reflect poorly on their brand reputation.

Participants from a range of organisations reported that demonstration of financial risks (regulatory, reputational, legal etc.) was a core driver of responsible investment activities. Correspondingly, several participants mentioned that, where the financial risks surrounding nutrition and obesity could be demonstrated, this was likely to be effective in stimulating investment action on these issues. Participants indicated that ‘what if’ scenario analysis projections were extremely useful for framing financial risks to investors. In particular, sugar taxation or other government action on obesity was said to provide a powerful example of potential real-world financial risk that could be estimated for food and beverage companies.

*“I think people get that nutrition and obesity is a social issue, and potentially a government health issue. You’ve then got to connect it through to why does it impact [particular companies]? That’s really the key thing for getting investors engaged… ‘What does that mean to how I value this company and whether this company is a good investment or not?’ ‘What are [the risks]?’ That’s really the key.” – Participant (ESG advisory/consultancy firm)*.

### Investment sector

A number of participants from investment organisations, ESG research and ESG advisory/consultancy firms and NGOs identified shortcomings with ESG data and interpretation as substantial challenges facing the responsible investment sector. Similarly, the lack of quality benchmarks and ESG data on issues related to food systems was seen as a barrier to the consideration of issues related to nutrition and obesity prevention within investment decisions. Participants reported that there were a multitude of ESG data providers and inconsistencies across ESG data and metrics led to heterogenous ESG ratings across companies. For example, companies that participants perceived to be ‘problematic companies’ (e.g., those that manufacture harmful products, such as tobacco) were sometimes given high ratings within commonly-available ESG datasets. This was because the ESG data provided by prominent ESG research providers could assign high scores to different ESG areas (such as aspects of corporate governance), without considering the nature of a company’s core business operations.

*“The FTSE100 showed the top five companies according to ESG rankings, and number three was British American Tobacco [a tobacco company]. It’s because they’re not considering health when they do those rankings. So [British American Tobacco] are very good on human rights and they do some environmental stuff and that’s why they’re up there. They’re obviously not getting scored down on health credentials, because, you know, investors just aren’t thinking about that at the moment.” – Participant (NGO)*.

As such, several participants reported that there was a need for increased knowledge on what ‘best practice’ looks like when it comes to investing in food companies. Participants wanted to understand how to compare and measure the ESG impact and exposure (with regards to nutrition and sustainability) of food companies. In particular, they noted that it was important to identify relevant metrics and benchmarks against which investors could assess their portfolios.

*“Yeah, so defining … what ‘good’ looks like [from an investor point of view is important], because I think once we can see that, then we can see what we have in our portfolios and see the gap between ‘good’ and what we have and we can start actually having some story telling around that. But without someone telling us what that end point looks like, it’s really hard.” – Participant (investment industry group)*.

Opportunities to facilitate responsible investment at the sectoral level included crises (e.g., COVID-19, natural disasters) or systemic issues (e.g., climate change) which were seen as triggering action due to their widespread negative impacts on different industries and economies. As such, a number of participants from a range of organisations noted that highlighting the negative systemic impacts that unhealthy food systems create and the links to climate change would be important for enabling consideration of these issues. Participants also reported that COVID-19 was likely to be a potential entry point for discussing health with the investment sector:

*“…our food systems are such a big issue and with this whole coronavirus thing and the questions we have to ask about food production and our global food systems and the risk that they’re posing, I think there is an opportunity to get it [nutrition and food systems issues] ‘in the door’ sort of from that global health and safety perspective. I think you have to be talking about linking it, because the link is there, to existential threats that our current food system creates.” - Participant (asset management organisation)*.

Some participants noted that the majority of investment action on food systems-related issues to date had come about through collaborative engagements between investors and advocacy groups. A prominent example of this in relation to nutrition was the Access to Nutrition Initiative (ATNI), which brings together over 70 institutional investors who can participate in collaborative engagements with food companies on nutrition issues [38]. Participants identified there were likely to be benefits to scale from exercising voice through collective action, including increased visibility of the issue and more pressure to respond. For example, one participant noted that food companies were more likely to be receptive to change where multiple investors raised the issue.

### Government

In the Australian context, a small number of participants reported that a challenge faced by the responsible investment sector was existing regulatory constraints on the investment activities of superannuation funds. Some participants from superannuation funds discussed the legal barriers to incorporating ESG-related considerations within traditional superannuation fund options. They noted that the underlying legal duty to act in the best (financial) interests of fund members may impede the integration of some ESG considerations in investment decision-making, particularly where ESG issues, potentially including nutrition- and obesity-related issues, were difficult to quantify as financial risks.

A small number of participants from superannuation funds and ESG research providers indicated that regulatory constraints were exacerbated by the perception that the Australian Liberal Government’s stance at the time was not supportive of activist investment and engagement on ESG risks (e.g., climate change):

*“I mean the government at the moment is quite vocal against, well any form of engagement on climate change risk… so there’s a lot of political process to manage as well. They’re [the Australian Government] pushing pretty hard against any sort of [ESG] values implementation in anything.” – Participant (superannuation fund)*.

For issues relate to nutrition and obesity prevention specifically, participants with broad insight into the investment sector (i.e., from investment industry groups and ESG advisory/consultancy firms) felt that there had been a lack of government leadership and national vision regarding efforts to improve the health and sustainability of food systems. Correspondingly, one of the primary opportunities to stimulate greater action on responsible investment in Australia reported by participants was legislation and regulatory changes regarding issues related to nutrition and obesity prevention (e.g., sugar taxation). Participants highlighted other ESG issues where regulation had been a driver of change, citing the example of the introduction of the Modern Slavery Act in 2019, which required large businesses to regularly report modern slavery risks in their operations and supply chains and actions to address those risks.

International developments, such as the European Union (EU) Green Deal (which will mobilise €1 trillion of sustainable investments over the next decade, towards a carbon neutral Europe by 2050 [52]), were seen as potentially foreshadowing regulatory changes in Australia, therefore prompting some investors to act. Consequently, one participant from an investment industry group noted that a regulatory framework governing sustainable finance activities (including requirements to disclose how ESG considerations are incorporated in investment decisions and the way in which finance is allocated to a set of agreed sustainability objectives) would likely help facilitate the consideration of obesity-related issues within investment.

### Non-government organisations

A number of participants from investment organisations with ethical values, ESG research providers and ESG advisory/consultancy firms highlighted the crucial role that vocal advocacy plays in driving consideration of ESG issues in investment decision-making. Media coverage of particular issues and associated public opinion was seen by some participants as an important lever for change, as was the level of controversy associated with an issue. Participants also highlighted the importance of watchdog and advocacy groups for ensuring accountability on food systems-related issues in Australia, noting the success of campaigns on animal welfare:

*“Animal welfare is the most likely entrée into the debate [around healthy and sustainable food systems] for responsible investors, mainly because there are well organised activist organisations who are now sophisticated enough to identify and target people [institutional investors] investing in companies associated with animal cruelty, particularly intensive farming and live animal export.” – Participant (ESG research provider)*.

For obesity-related issues specifically, several participants felt that there was limited advocacy and groundswell on addressing the health of food systems in Australia, and limited NGO resourcing for addressing and advocating for these issues. Participants from NGO’s noted that advocacy campaigns targeting food companies may be more likely to gain investor support if they adopted a less combative style and were not perceived as ‘demonizing’ food companies.

## Discussion

This study identified a range of challenges and associated opportunities for increased consideration of issues related to nutrition and obesity prevention within institutional investment decision making. These included challenges and opportunities at the investor employee level (e.g., experience and training, internal pressure/buy in), investment organisation level (e.g., competing duties and issues, extent of topic-specific knowledge, investment ethos and approach, exposure to food companies, member/client demand, brand reputation, demonstration of financial risks), investment sector level (e.g., quality and availability of ESG data, focus on systemic issues/crises, exercising voice through collective action), government level (e.g., regulatory measures and legal constraints, resourcing and leadership) and non-government level (e.g., media attention and public opinion, advocacy).

These findings are similar to recent research by the group ShareAction (a UK based charity that promotes responsible investment and improvements to corporate ESG practices) involving institutional investors in the UK, which showed that investor stewardship on health issues is lacking [53]. ShareAction reported that in order to support the integration of health within investment practices, there was a need to further demonstrate the ‘business case’ for prioritising health, develop evidence-informed guidance on topics and sectors to focus on, as well as high quality data on company performance [53]. Below, we discuss key recommendations for action identified from this study, with a focus on what is needed to help embed considerations related to nutrition and obesity prevention within institutional investment decision-making in Australia.

### Improve the quality and availability of nutrition related ESG data and benchmarking criteria

One of the main challenges identified through this study for investors to consider issues related to nutrition and obesity prevention, and for responsible investment decision-making in Australia more broadly, were limitations in available ESG data and associated benchmarking criteria. Participants from a range of organisations reported using different data sources to inform their decision making, including in-house data analytics, third-party ESG data, benchmarks and corporate reporting. Inconsistencies in the methodologies across ESG data providers and in-house data analytics was seen as diminishing the comparability of ESG performance across companies. Heterogenous ESG data is a commonly cited problem across the global investment sector [54, 55]. In 2019, State Street Global Advisors assessed the extent of ESG rating variation across major ESG ratings providers (including Sustainalytics, MSCI, RobecoSAM, Bloomberg ESG) and found that there was substantial differences in research, data sources and scoring methodologies [54]. An academic study comparing the ESG rating approaches of MSCI, Bloomberg and Thomson Reuters datasets found that, while dimensions of ESG were similar, the composition and weightings of indicators led to substantial differences in overall ESG scores assigned to particular companies, particularly in the case of the ‘social’ domain [56]. Thus, a key driver of investor action on issues related to nutrition and obesity prevention in Australia is likely to be comprehensive and consistently applied ESG ratings for food companies that include nutrition-related metrics.

Corporate sustainability reporting is one of the primary data sources used by ESG data providers and investors to assess companies on their ESG performance. There is a lack of globally agreed reported metrics regarding ESG issues, and international frameworks and guidelines differ markedly [57]. Five prominent framework- and standard-setting institutions have recently committed to work together towards implementing a comprehensive corporate reporting system, which is an important step towards consolidation of sustainability reporting standards [58]. However, previous research suggests that the incorporation of nutrition and obesity-related indicators within global ESG reporting standards and frameworks is limited in scope, with inconsistent application [47, 59]. Moreover, disclosure of nutrition- and obesity-related policies and practices as part of corporate reporting has been shown to be limited and highly variable across the food industry [60–64]. Assessment of 350 influential food and agricultural companies as part of the 2021 Food and Agricultural Benchmark, conducted by the World Benchmarking Alliance (WBA), found that, out of ‘nutrition’, ‘environment’ and ‘social inclusion’ domains, nutrition had the smallest number of publicly disclosed commitments [60]. Until corporate sustainability reporting standards explicitly include nutrition-related criteria for food companies, there is unlikely to be widespread uptake of nutrition-related performance metrics by investors.

One of the key opportunities to support investor action on issues related to nutrition and obesity prevention, identified through this study, is to increase understanding of ‘best practice’ investment practices with respect to food companies. Study participants described benchmarking tools as useful for understanding how companies could be assessed against food systems-related issues. Prominent nutrition benchmarking tools include the Access to Nutrition Initiative (ATNI) [38], which benchmarks global food and beverage manufacturers on their nutrition-related practices and on the healthiness of their product portfolios, and the WBA Food and Agricultural Benchmark, which assesses food and agricultural companies on their performance against nutrition, social inclusion and environmental criteria [60]. While these initiatives do not include data and benchmarks for all regions and food companies, increased uptake of benchmarking and accountability initiatives, like the WBA Food and Agricultural Benchmark and the ATNI, is likely to help identify food industry leaders and laggards and address some of the literacy issues surrounding the application of nutrition- and obesity-related issues to an investment portfolio.

### Demonstrate the financial argument for addressing unhealthy diets and obesity

Another commonly cited challenge (and associated opportunity) to the incorporation of issues related to nutrition and obesity prevention within investment decision-making was the perception that the financial argument for investors to address these issues has not been clearly demonstrated. Nevertheless, study participants did highlight several material financial risks for investors, including those associated with reputational, regulatory and systemic risks for investee companies. In regards to reputational risks, a 2013 report by Credit Suisse on investment risks associated with sugar highlighted that a major risk for investors was negative public opinion and consumer awareness of the ‘sugar debate’ [65]. Such public sentiment and associated reputational scrutiny on food companies is likely to be a driver of increased action from institutional investors. Of note, participants reported that regulatory risks (such as those linked to the introduction of sugar taxes) were likely to be the most relevant source of financial risks for food sector companies and their investors. Estimates from a 2017 report by Schroders and Rathbone Greenbank Investments suggested that regulatory and consumer pressure on sugar could lead to an ‘earnings per share’ reduction of 3–25%, depending on a company’s exposure to unhealthy products [39]. A number of economic modelling studies have estimated costs to industry associated with implementing obesity-related regulation, for example, the UK Food Standard Agency’s Impact Assessment for voluntary reformulation estimated a cost to industry of £25,000 per product reformulated [66–68].

The regulatory context for obesity prevention in Australia has typically preferenced voluntary and industry-guided approaches [69]. Unlike many other countries, the Australian government has not committed to implement globally recommended policies such as a sugar sweetened beverage tax, mandatory interpretive front-of-pack labelling and restrictions on the marketing of unhealthy foods and beverages to children [70–72]. This may explain, in part, current investor approaches which do not treat nutrition and obesity prevention-related issues as material financial risks. The recent (2022) change of government in Australia [73] to a political party with more progressive values may influence the regulatory environment for obesity prevention in Australia, and, coupled with growing regulatory action and consumer pressure on food companies globally, could provide increased impetus for Australian investors to respond.

One of the key areas to mobilise investment for nutrition- and obesity-related issues suggested by participants was demonstrating the impact these issues can have on investments, for example through real-world impact assessments and scenario analyses that assess policy options or consumer trends. Further research into what this would look like in practice and how it could be presented in a useful way for investors is likely to be valuable. Whilst regulation for improved nutrition and obesity prevention was generally viewed by participants in this study as a financial risk in relation to food companies, such regulation may also be viewed as an opportunity, e.g., for companies with healthier product portfolios or through increasing potential earnings indirectly via a more productive workforce and healthier communities. Economic modelling of obesity prevention legislation in Australia and internationally, such as sugar taxation, mandatory reformulation and restrictions on marketing to children, overwhelmingly show that these are cost-saving interventions from a societal perspective, with enormous public benefits to health systems, governments and society over the long term [74–77].

Moreover, universal owners (with highly diversified investment portfolios), by their nature, are not able to diversify away from systemic risks (i.e. risks that effect the entire market or system) [78, 79]. Diet-related ill health is an issue that impacts companies across different sectors through loss in productivity and ill health-related absence from work (e.g., as evidenced through the COVID-19 pandemic) which has the potential to affect investors portfolios across the board [80]. Whilst some participants from institutional investment organisations in this study reported that they were actively thinking about ESG issues in a systemic way, many other participants reported that the financial sector in Australia is still considering ESG in simplistic and superficial terms, which is likely to affect the extent to which nutrition issues are viewed as material to various industries and sectors. Outside of productivity losses, there is evidence that tackling unhealthy diets and obesity can help to mitigate environmental sustainability risks and harness associated sustainability opportunities [1]. Climate change is widely considered one of the most pressing systemic risks facing the economy, and there is growing recognition amongst the financial sector that climate-related risks and opportunities apply to all sectors of the economy [79]. There is a substantial amount of research demonstrating the interconnectedness of unhealthy diets and climate change, with a recent Lancet report noting that “alongside curbing air pollution, greater adoption of healthy and sustainable diets is potentially the greatest synergy between human and planetary health” [8]. The current study showed that, while climate change was front-of-mind for investors, there appeared to be limited recognition among investors about the ways in which healthy and sustainable diets were correlated with climate change. Increased communication with the investment sector around the links between unhealthy and unsustainable diets may, therefore, be helpful in framing unhealthy diets and obesity in financial risk terms. Overall, increased understanding of the financial implications that unhealthy diets and obesity could have on an investment portfolio, and what this means for investors with shares in food companies, represents a real opportunity to drive further consideration of these issues.

### Build momentum around issues related to nutrition and obesity prevention in Australia through investor coalitions

This study highlighted the perceived importance of investor coalitions that use their collective voice to engage with companies on a particular issue, rather than relying on individual investor activism. Alongside corporate engagement, a recent report by the Food Foundation (a UK policy advocacy and research organisation) highlighted the largely untapped power of investor coalitions in influencing government action on food systems issues and engaging with policy decision-making [81]. Groups like the Food Foundation, ATNI and ShareAction are making progress in building investor coalitions that engage on food systems-related issues [44, 81–83]. ATNI has at least 74 investment organisations representing over USD$16.5 trillion assets under management that have signed on to the ATNI Investor Expectations on Diets, Nutrition and Health which outlines four investor expectations related to corporate governance, strategy, lobbying and transparency for food and beverage manufacturers and retailers [38, 84]. Importantly, because these types of initiatives frame their findings for an investor audience and encourage uptake by investors, they can mobilize multiple investors to engage with food companies on their obesity policies and practices. A recent example of this is an investor coalition organised by ShareAction, who filed shareholder resolutions at two major UK food retailers (Tesco and Morrison’s) asking them to increase sales of healthy products whilst publicly reporting on targets and progress [43]. Advocacy was highlighted by participants in this study as one of the key factors that contributed to the success of other investor targeted initiatives related to food systems, for example those focused on animal welfare and intensive animal agriculture. In particular, advocacy and engagement conducted by the Farm Animal Investment Risk and Return (FAIRR) initiative was seen as successful in building investor momentum on ESG risks and opportunities related to intensive livestock production [85].

There are, however, only a small number of Australian based investors (AMP Capital, Apostle Funds Management, Christian Super, Ethical Partners Funds Management, Local Government Super) who are signatories to the ATNI Investor Expectation on Diets, Nutrition and Health [83]. This could be due to the type of companies included in the flagship ATNI benchmark, none of which are Australian-listed companies. Additionally, unlike in the UK where ShareAction and the Food Foundation are active, there is no investor coalition that is targeted towards addressing nutrition, obesity or food systems issues in Australia. The creation of such a coalition in Australia may help to generate interest in addressing issues related to nutrition and obesity prevention amongst the Australian institutional investment sector, particularly where there is low awareness or engagement with the activities of internationally-based nutrition initiatives. There may also be substantial opportunity for advocacy organisations operating in the public health space (e.g., Obesity Policy Coalition, Cancer Council, Public Health Association of Australia) to scale up engagements with the institutional investment sector as a stakeholder in efforts to improve population diets and prevent obesity, including as part of advocacy efforts that aim to influence government policy decision-making.

### Provide guidance on ways that institutional investors in Australia can address unhealthy diets and obesity

A number of participants reported that one of the primary impediments to increased attention to issues related to nutrition and obesity prevention was the lack of opportunities to invest in food companies on the Australian Securities Exchange (ASX). Interviewees whose organisations invested in predominantly Australian equities therefore perceived obesity as a less relevant ESG issue for their portfolios. These findings echo commentary from EIRIS (now Vigeo-EIRIS), an international provider of ESG research and services, that noted in 2016 that “in the Australian S&P/ASX 300 index there are only three companies that have been categorised with high obesity exposure” [86]. Moreover, the focus on ‘obesity’ by investors was perceived to be centred around large multinational food and beverage companies. Whilst many of the investors in this study invest in these multinational food companies, engagement efforts were predominantly concentrated on local companies due to a range of practical reasons. Investor coalitions could a play a key role here in driving engagement with internationally-listed companies on the issue of obesity and nutrition. In particular, multinational investor coalitions, such as those coordinated by ATNI and ShareAction, may help to facilitate Australian investor participation in collaborative ESG engagements with internationally-listed food companies. Furthermore, multinational investor coalitions may provide an opportunity for Australian investors to co-sign health-related shareholder resolutions filed at these companies. Increasing Australian investment sector awareness of, and participation in, these types of coalitions is likely to provide investors with more international corporate engagement opportunities.

Furthermore, while these findings would suggest that there are fewer opportunities to invest and engage with food companies in Australia, there is likely to be substantial room for improvement on nutrition- and obesity-related performance for those food companies that are listed on the ASX, particularly the largest Australian food retailers. EIRIS noted that no listed Australian companies scored well in regard to their efforts to address obesity [86]. Recent assessment of the nutrition and obesity prevention-related policies and commitments of major food retailers in Australia (Woolworths, Coles, Metcash/IGA and ALDI) found that they all performed relatively poorly on obesity prevention efforts [62]. Moreover, assessment of Woolworths and Coles in the WBA Food and Agricultural Benchmark found that neither company had “a comprehensive set of commitments to help consumers make healthier choices” [60]. These findings point to the need for increased accountability of Australian food retailers in addressing obesity and improving population diets, and indicate a number of areas for improvement in which Australian institutional investors could engage.

Difficulties in understanding the relevance of nutrition-related issues to companies outside of the food industry was identified by some participants as impeding the consideration of issues related to nutrition and obesity prevention within investment decision making in Australia. There are many companies outside the food industry that are likely to have the capacity to take action on nutrition through initiatives such as staff wellbeing programs, individual nutrition education and nutrition standards for food provision (e.g., through internal catering, food procurement and provision policies). Whilst these types of initiatives can contribute to obesity prevention efforts [87], investors are, arguably, likely to have the most influence on population diets where efforts are directed towards food and beverage industries and actions that alter the healthiness of food environments at the population level. This is evidenced by the demonstrated large population health benefits and cost-effectiveness of initiatives that impact food industry policies and practices, such as sugar sweetened beverage taxation, restrictions on marketing of unhealthy foods, and product reformulation [75], coupled with the relatively lower population health benefits from education-based initiatives [88]. Nevertheless, evidence of the impact of workplace-level nutrition-related actions on population health outcomes and productivity warrants increased attention. Outside of diets and nutrition, investors may be able to consider ‘obesity’ more broadly through their investment in healthcare, obesity treatment and management, and increasing attention to employee health and wellbeing [80]. Although this was not explored in the current study, broader strategies for investors to consider obesity-related issues may provide an additional avenue for nutrition and obesity prevention to gain traction as an ESG issue.

### Strengths and limitations

This is the first study that we are aware of that qualitatively explores stakeholder perspectives on institutional investment related to obesity. This study builds on the findings from a previous desk-based study investigating the incorporation of nutrition within investment decision-making by leading institutional investors in Australia, to provide more in-depth understanding of challenges and opportunities for institutional investors in this area. The sample of participants included a number of stakeholders with extensive and diverse experience working within the investment sector. This study used an established theoretical framework to guide data analysis, which is likely to have enhanced the rigour of the findings and increased the likelihood that the findings are transferrable to other contexts.

This study has several limitations. Whilst a broad range of participants (with different areas of interest and expertise) were involved in the interviews, those willing to participate in the research may have had more of an interest in responsible investment or addressing obesity-related issues within investment decision-making than the broader investment community. Thus, some participation bias may have been introduced. This study focused on the perspectives of the Australian investment sector. It would also be valuable if future research encompassed food industry perspectives regarding the potential influence of investors on food company practices and performance. Another limitation of this study was its relatively small sample size (15 interviews). The characteristics of the participant sample may mean that some of the findings are less generalisable to investors who are not engaged in responsible investment. Nevertheless, the extensive industry expertise of the participants, and the wide-ranging discussion of challenges and opportunities, indicate that the findings are likely to be highly relevant to the institutional investment sector more broadly and represent an important contributin to the literature. The paper focused on concerns related to obesity prevention, with a focus on population nutrition and, particularly, investment in food companies. The paper did not consider in detail: company initiatives related to employee wellbeing (e.g., staff wellness programs); considerations related to physical activity; or obesity treatment and management. We also did not focus on broader nutrition aspects, such as efforts to address under-nutrition, food security, and the environmental sustainability of food systems. The potential for the investment community to consider each of these aspects warrants further investigation. Due to the qualitative nature of this study and the fact that the majority of stakeholders were based in Australia, the applicability of the findings to other countries where regulatory and financial market contexts differ from Australia may be limited. However, the global nature of financial systems and the obesity-related ESG issues examined, also suggest that our findings will be broadly relevant.

## Conclusion

Unhealthy diets and obesity are among the most pressing public health issues in Australia and globally, and present growing risks to a healthy and sustainable society and economy. Improvements to food industry policies and practices related to nutrition, coupled with efforts to increase the accountability of the food industry for their role in producing and marketing unhealthy products, is an important component of efforts to improve population diets and address obesity. Institutional investment that incorporates ESG considerations related to nutrition and obesity prevention is a potential avenue for increasing food industry action and accountability in this area. The findings from this study indicate that, despite apparent commitment to ESG factors as part of decision-making in Australia, there appears to be only limited consideration of nutrition and obesity prevention as part of current ESG frameworks. The limited consideration of nutrition and obesity prevention indicates that the investment case for action on unhealthy diets and obesity may not be clearly defined in the Australian context. More broadly, the lack of comprehensive investor action in this area likely illustrates the broader limits to the types of ESG-related actions many institutional investors are willing to take within the regulatory and institutional systems in which they currently operate.

This study identified a range of recommended actions that could help ensure more systematic and effective consideration of nutrition- and obesity-related issues within institutional investment decision-making in Australia, including: (1) improved nutrition-related reporting metrics and benchmarking criteria for food companies; (2) better articulation of the financial risks that unhealthy diets and obesity pose to investors; (3) enhanced investor advocacy on unhealthy diets and obesity through investor coalitions; and (4) detailed guidance for investors on how to address unhealthy diets and obesity. Better engagement between the Australian public health community, institutional investors and government regulators is critical to drive changed investor practice in this area.

## Electronic supplementary material

Below is the link to the electronic supplementary material.

Supplementary Material 1

Supplementary Material 2

## Data Availability

The datasets analysed during the current study are not publicly available due to this study reporting qualitative data collected from stakeholders working within the investment sector. Qualitative interview data is by nature difficult to completely de-identify. Ethical approval for this project stated that participants’ data would only be accessible by the research team.

## Abbreviations

ASX: Australian Securities Exchange
ATNI: Access to Nutrition Initiative
AUD: Australian Dollars
CSR: Corporate Social Responsibility
ESG: environmental social governance
EU: European Union
FAIRR: Farm Animal Investment Risk and Return
LSE: London Stock Exchange
NCD: Non-communicable disease
NGO: Non-government organization
RIAA: Responsible Investment Association of Australasia
SDG: Sustainable Development Goal
UK: United Kingdom
WBA: World Benchmarking Alliance.
