# Supplementary material for: Integrating nutrition and obesity prevention considerations into institutional investment decisions regarding food companies: Australian investment sector perspectives
Source: Global Health. 2022 Nov 8;18:93. doi: 10.1186/s12992-022-00885-7 (PMC9640902; doi:10.1186/s12992-022-00885-7)
Supplement: Supplementary file 1 — Supplementary Material 1 [file 12992_2022_885_MOESM1_ESM.docx]

**Additional file 1: Interview schedule**

**Open**

*Introduction and purpose of interview.*

**Questions**

1. To start, could you please tell us about your role and your involvement in responsible/ethical investment?
2. To what extent does the issue of healthy or environmentally sustainable food systems come up in your work in relation to responsible investment?
   - Prompt: In what ways does it tend to come up?
   - Prompt: Are their particular dimensions of healthy and sustainable food systems that come up most often (e.g., sugar, obesity, animal welfare, deforestation related to agriculture etc.)
   - Prompt: Why do you think that these particular issues get more attention? What do you think has driven this?
   - Prompt: Is that a more recent thing that these issues have been talked about or has that been in the works for a while?
3. To what extent do you think the responsible investment community considers issues related to healthy and sustainable food systems in investment decision making?
   - Prompt: Are these issues considered more in some parts of the industry? If so, where?
4. Are issues related to social justice in the food sector considered, such as slave labour or wage theft? If so, how and by whom?
5. What are the drivers for consideration of healthy, sustainable food system issues in investment decision making?
6. Where issues related to healthy and sustainable (or socially just) food systems are considered in responsible investment, what types of data or metrics are used?
   - Prompt: What are the sources of the data or metrics?
7. To what extent do companies report against issues related to healthy and sustainable food systems?
   - Prompt: How do they do this?
   - Prompt: Discuss more on internal vs external reporting, PRI, mandatory reporting
8. For those investors that are considering these issues, where do you think these sit on the priority list?
9. Are there opportunities for responsible investment to focus more on food-related issues to help drive a transition to healthy and sustainable food systems?
   - Prompt: What would need to happen in order to realise those opportunities?
   - Prompt: Are particular sections of the RI community showing more appetite than others?
10. Where might there be most scope for the expansion of responsible investment that positively supports healthy and sustainable food system considerations, and/or divestment from industries and companies that negatively impact these considerations?
11. What would you say are the key barriers to incorporating these issues into responsible investment in Australia?
12. Can you think of any ways that we as researchers could help to support these issues being incorporated into responsible investment?

**Close**
