# Supplementary material for: Integrating nutrition and obesity prevention considerations into institutional investment decisions regarding food companies: Australian investment sector perspectives
Source: Global Health. 2022 Nov 8;18:93. doi: 10.1186/s12992-022-00885-7 (PMC9640902; doi:10.1186/s12992-022-00885-7)
Supplement: Supplementary file 2 — Supplementary Material 2 [file 12992_2022_885_MOESM2_ESM.docx]

**Additional file 2: Coding framework**

| Theme (actor) according to theory^1^ | Theme (actor) in this study | Sub-theme (mechanisms) according to theory^1^ | Sub-theme in this study |
| --- | --- | --- | --- |
| Employees | Employees within institutional investment organisations | Pressure to increase level of CSR  Employee participation and leadership  Organisational commitment  Job satisfaction  Employee citizenship  Performance | Experience and training  Internal pressure/buy in |
| Stakeholders (insiders) | Institutional investment organisations | Direct strategic decisions | Competing duties and issues  Extent of topic-specific knowledge  Investment ethos and approach  Exposure to food companies  Member/client demand  Brand reputation  Demonstration of financial risks |
| Stakeholders (outsiders) | Investment sector | Exercising voice through collective action | Quality and availability of ESG data  Focus on systemic issues/crises  Exercising voice through collective action |
| Intergovernmental organisations |  | “Bully pulpit”  Policy papers |  |
| Domestic governments | Governments | Law enactment  Law enforcement  “bully pulpit”  Education of best practices | Regulatory measures and legal constraints  Resourcing and leadership |
| Non-governmental organisations | Relevant non-government organisations | Campaigns and boycotts  Multiparty dialogue | Media attention and public opinion  Advocacy |

^1^Social change theoretical model, originally developed by Aguilera et al. 2007 (27)
